# Advances in surface modifications of the silicone breast implant and impact on its biocompatibility and biointegration

**DOI:** 10.1186/s40824-022-00314-1

**Published:** 2022-12-14

**Authors:** Fatemeh Tavakoli Foroushani, Kevin Dzobo, Nonhlanhla P Khumalo, Vanessa Zamora Mora, Roberto de Mezerville, Ardeshir Bayat

**Affiliations:** 1 Wound and Keloid Scarring Research Unit, Hair and Skin Research Laboratory, Division of Dermatology, Department of Medicine, The South African Medical Research Council, University of Cape Town, Cape Town, South Africa; 2 Establishment Labs Holdings, Alajuela, Costa Rica

**Keywords:** Silicone breast implants, Foreign body response, Capsular contracture, Biocompatibility, Silicone surface modification

## Abstract

Silicone breast implants are commonly used for cosmetic and oncologic surgical indications owing to their inertness and being nontoxic. However, complications including capsular contracture and anaplastic large cell lymphoma have been associated with certain breast implant surfaces over time. Novel implant surfaces and modifications of existing ones can directly impact cell-surface interactions and enhance biocompatibility and integration. The extent of foreign body response induced by breast implants influence implant success and integration into the body. This review highlights recent advances in breast implant surface technologies including modifications of implant surface topography and chemistry and effects on protein adsorption, and cell adhesion. A comprehensive online literature search was performed for relevant articles using the following keywords silicone breast implants, foreign body response, cell adhesion, protein adsorption, and cell-surface interaction. Properties of silicone breast implants impacting cell-material interactions including surface roughness, wettability, and stiffness, are discussed. Recent studies highlighting both silicone implant surface activation strategies and modifications to enhance biocompatibility in order to prevent capsular contracture formation and development of anaplastic large cell lymphoma are presented. Overall, breast implant surface modifications are being extensively investigated in order to improve implant biocompatibility to cater for increased demand for both cosmetic and oncologic surgeries.

## Background

Breast augmentation is one of the most common plastic surgical procedures performed to correct breast volume and shape abnormality for both cosmetic and oncologic indications. Estimates put the number of breast implants at around 8 million worldwide [1]. According to the International Society of Aesthetic Plastic Surgery (ISAPS) statistics report of 2019 [2], approximately half of the total annual breast procedures in the world were breast augmentations, with 89% using silicone implants [2]. Since 1963, silicone breast implants have been utilised increasingly for both plastic and reconstructive surgical indications. Indeed, significant improvements have been observed in terms of their biocompatibility and safety [3, 4]. For example, Choi and colleagues demonstrated that the presence of micro-textures on silicone implants suppress foreign body response (FBR) and capsular contracture [5]. However, significant problems continue to exist, and various strategies have been utilised to improve silicone implant performance which continues to remain under investigation. These strategies are aimed at improving implant biocompatibility and enhancing implant integration in the human body as well as the prevention of silicone implant ‘bleeding’ and stimulation of FBR [6, 7].

Silicone, with the most common being polydimethylsiloxane (PDMS), has many diverse uses including incorporation in medical devices such as implants and catheters [8, 9]. As an inert material, silicone does not degrade and displays great properties ranging from low toxicity, as well as being gas permeable [10, 11]. Despite its appealing properties, silicone materials also exhibit a hydrophobic nature [12]. Silicone hydrophobicity leads to poor anti-fouling activities and this can impact on its efficacy in medical devices [13]. Implantation of ‘foreign material’ in the body causes the host tissue to initiate a defence mechanism known as FBR, characterised by a series of humoral and cellular activities. The extent of host response depends on several factors including the foreign material’s biocompatibility and the host tissue’s response.

Adsorption of proteins onto the implant surface facilitates the attachment of various cells including immune cells and fibroblasts as well as the activation of signalling cascades leading to formation of a capsule around the implant. Excessive FBR may result in enhanced extracellular matrix (ECM) synthesis leading to the formation of a dense contracting capsule which prevents the proper implant-breast tissue integration ultimately leading to structural deformation of the implant and continuous pain and discomfort to patients [12, 14]. This necessitates the replacement of the old implant. Implant surface properties play key roles in the adsorption of proteins and ultimately the attachment of cells to the implant. Thus, a large portion of research has been dedicated to investigating implant surface properties and modifications and effects on integration into the body [15–22]. For instance, of note macro-textured silicone breast implants have been linked to the development of anaplastic large cell lymphoma (ALCL) [1].

Several *in vitro* and *in vivo* studies on silicone breast implants have revealed insight into the role of implant surface in mediating cellular and tissue behaviour or function (Table 1) [15–24]. These studies demonstrated that implant properties including topography can influence implant integration and their ultimate fate *in vivo* [17–20, 22]. A number of studies have shown that breast-derived fibroblasts are affected by the physical and chemical characteristics of the silicone shells and over time adapt to the specific topographies of the material after being stressed [25–27]. Interestingly, in an *in vitro* study carried out by Kyle et al., the effect of silicone implant surfaces compared with biomimetically engineered silicone surfaces was evaluated using breast-derived fibroblasts [18]. This study demonstrated that a biomimetic-engineered implant surface was superior in attenuating FBR in terms of development of an inflammatory response compared with most common commercially available silicone implant surfaces [18]. Furthermore, Doloff et al. studied the effect of silicone breast implant surface topography on capsule formation and FBR in human and other animal models [15]. The authors discovered, using both animal and human studies, that biomimetic surfaces with the roughness of around 4 μm provoked the least amount of inflammation as well as FBR. In another study using both animals and humans, Chung and colleagues were able to identify immune regulators of FBR after implantation of implants including silicone breast implants [21]. An *in vivo* study by Brigaud and co-workers investigated ECM and inflammatory gene expression in capsules from human patients using textured implants and showed that implant surface topography provide ‘cues’ that impact gene expression [28].

**Table 1:** Studies involving cell- breast implant interactions using both *in vitro* and *in vivo* methods

Aim of study	*In vitro*	*In vivo*	Implant surface	Characterisation	Ref
	**Cells**	**Animal/** **human**			
Effect of different silicone topographies on breast normal fibroblast reaction and its orientation	Breast-derived fibroblast	-	-	Light microscopy, immunofluorescent assay, and atomic force microscopy	[20]
Investigation of the interactions between fibroblast and different silicone breast implant surfaces	Skin fibroblast	-	Smooth and textured	Cell detachment induced by trypsin	[19]
The effect of botulinum neurotoxin type A on capsule formation around silicone implants	Skin fibroblast	Mice	Smooth	Histologically analysis of fibrotic capsules surrounding the implant, immunofluorescence microscopy, quantitative polymerase chain reaction analysis of cytokine expression cell attachment and proliferation assay, western blot analysis, and enzyme-linked immunosorbent assay	[24]
Comparing silicone implant surfaces with biomimetic silicone surfaces with hierarchical micro/nano-topographical features	Breast derived fibroblast	-	Smooth and textured	Cell attachment, proliferation, cytotoxicity assay, immunofluorescence microscopy, Scanning electron microscopy, quantitative polymerase chain reaction analysis, and cytokine array	[18]
Assessment of a range of commercially available breast implants in terms of biocompatibility	THP-1 macrophage	-	Smooth and textured	Immunocytochemistry analysis, immunofluorescence microscopy, Scanning electron microscopy, quantitative polymerase chain reaction analysis, and inflammatory marker cytokine array	[17]
Clarification of the relationship between periostin and the process of capsule formation after *in vivo* implantation	-	Mice	Smooth	Histologically analysis of fibrotic capsules, immunohistochemical analysis, and western blotting	[16]
Investigation of the immune response of human peripheral blood mononuclear cells (PBMC) to silicone breast implants	Blood mononuclear	-	Smooth and texture	Cell proliferation assay, quantitative polymerase chain reaction analysis, and multiplex Immunoassay	[23]
Assessment of surface texture of commercially available breast implants in host tissue response	-	Rat	Smooth and textured	Scanning electron microscopy, X-ray computed tomography, and histologically analysis of fibrotic capsules	[22]
Identify adaptive immune regulators of the FBR to synthetic material implants including silicone breast implants	-	Mice and human	Not mentioned	Histologically analysis of fibrotic capsules, quantitative polymerase chain reaction analysis, flow cytometry analysis, cell proliferation assays, immunofluorescence assay, IL17 neutralisation, IL6 blocking antibody, and senolytic treatment	[21]
Effect of the surface topography of silicone breast implants on the FBR	-	Mice, rabbit, and human	Smooth and textured	Histologically analysis of fibrotic capsules, immunofluorescence microscopy quantitative polymerase chain reaction analysis, FACS analysis, and nanoString analysis	[15]
Determine whether implant surface topography can affect the physiology of asymptomatic capsules		Human	Textured	Investigated ECM and inflammatory gene expression in human capsules from patients using textured implants	[28]

The aim of this review is to present recent advances in implant surface technology including surface topography and chemistry, and to demonstrate how these impact silicone implant biocompatibility and biointegration. We demonstrate that roughness and wettability influence the type and number of proteins adsorbed onto the implant surface and therefore can affect the FBR. Overall, we show that modifications of implant surfaces are important in enhancing biocompatibility.

## Methodology

We performed an electronic search on Scopus, PubMed and Web of Science to identify articles to be included using specific search strings including keywords silicone breast implants, host response, cell adhesion, protein adsorption, and cell-surface interaction. This comprehensive literature search was performed for relevant manuscripts on the evolution of breast implants and origins of theories. Abstracts and titles of studies were screened by authors and included if they met the inclusion criteria. Duplicate articles were removed.

## Past and current silicone-gel breast implants used in surgical practice

**Fig. 1:**
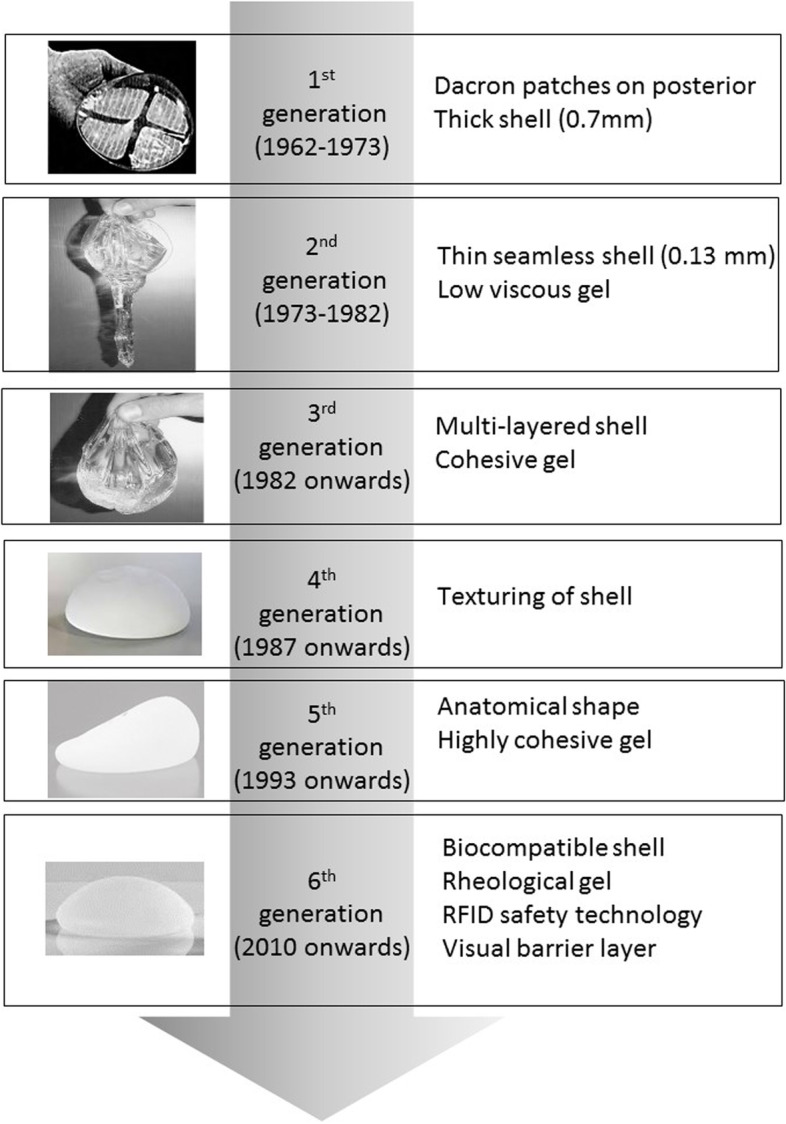
Historical evolution of silicone breast implants. Important breakthroughs in silicone implants technologies starting with implants with Dacron patches on the posterior side to the latest implants with biocompatible shells and radio frequency identification technologies. First generation silicone breast implants were made from thick smooth silicone elastomer shell filled with Silastic. Second generation implants consisted of thinner and seamless shells, with the fixation patches removed. Third generation implants were introduced from 1982 onwards and consisted of high-performance multi-layered shells with a cohesive gel. The introduction of texturing saw the entrance of fourth generation implants from 1987. From 1993, fifth generation implants were introduced, and these have an anatomical shape with a highly cohesive gel. From 2010, sixth generation implants were introduced, and these have a biocompatible shell and a rheological gel. Other technologies such as radio frequency identification are associated with sixth generation silicone implants

Silicone gel implants have been used widely since the 1960s (Fig. 1). Interestingly, the first generation of silicone-gel breast implants consisted of a teardrop pre-shaped prosthesis composed of a thick (0.75 mm), smooth silicone elastomer shell filled with Silastic™ and a dacron mesh backing, which became anchored in the underlying pectoral fascia [29]. Due to high cases of capsular contracture and firmness of these original silicone gel-filled implants, new types of silicone-gel filled implants emerged, with thinner (0.13 mm) and seamless shells, which were introduced in 1970s. The new silicone gel filled implants had low viscosity to provide a more natural feel, and the fixation patches were eliminated [30]. However, several reports showed that these second-generation implants suffered from increasing firmness, shell rupture and bleeding of the silicone gel [31, 32]. Therefore, third-generation implants with high-performance multi-layered shells and a much more cohesive gel were introduced [33, 34].

Aiming to prevent the development of capsular contracture, double-lumen implants consisting of an inner silicone gel-filled lumen surrounded by an outer saline inflatable shell were designed [35]. In addition, the reverse type of implants, with an outer silicone gel-filled shell surrounded an inner inflatable shell, have been investigated. The reverse double-lumen was shown to provide better properties including no wrinkling of the outer shell, a more natural breast feel, and a nonpalpable self-sealing posterior valve compared to the inflatable saline-filled breast [36]. Concerns related to biocompatibility and safety use of silicone gel implants led to provisional restrictions on some specific implants from the American market in 1992 by the Food and Drug Administration (FDA) until 2006. In turn, core studies to assess overall implant safety profiles have been carried out [37–40] and led to development of silicone gel-filled implants focusing on shell properties, gel cohesiveness, and anatomical shape.

## Breast tissue response to implant

The insertion of an implant into the breast tissue results in an inflammatory reaction by the body referred to as FBR which is characterised by the release of immune factors such as chemokines and cytokines. Enhanced levels of chemokines and cytokines result in excessive inflammation as well as increased synthesis of several ECM proteins [41, 42]. This section briefly describes the breast tissue wound healing response to the placement and presence of the implant.

### Breast anatomy and histology: implications on implant placement

**Fig. 2:**
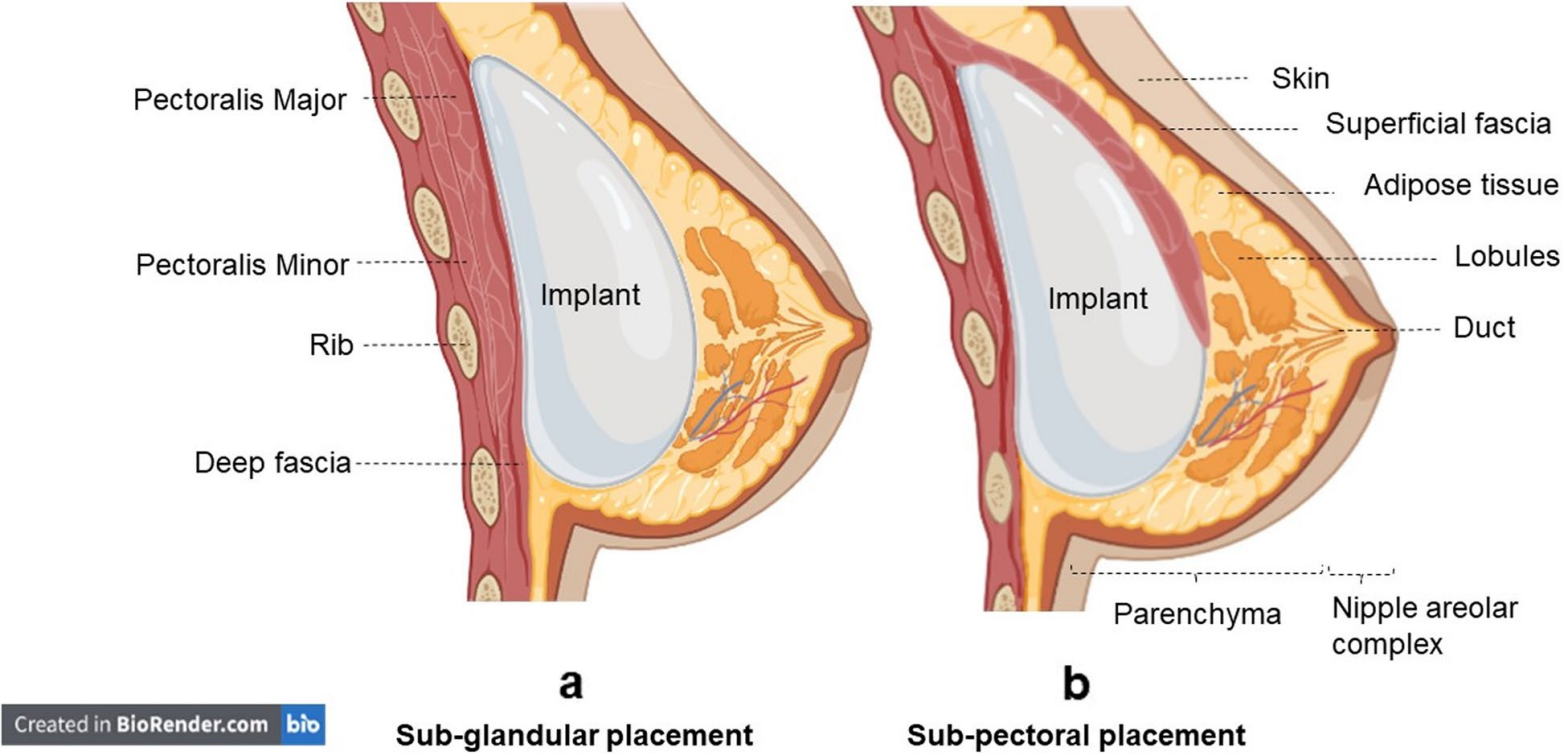
Breast anatomy and implant placements: **a** Sub-glandular placement, **b** Sub-pectoral placement. An incision (infra-mammary, peri-areolar, trans-axillary, or trans-umbilical) is made to place the implant either in the sub-glandular (**a**), dual-plane/ partial under the muscle, or sub-pectoral (**b**)

The human breast is composed of five components: 1) skin 2) superficial fascia, 3) breast parenchyma 4) nipple (areola complex) and 5) deep fascia [43] (Fig. 2). The parenchyma can be divided into three tissue types: glandular epithelium, fibrous stroma, and supporting structures and fat (adipose tissue) [44]. The glandular epithelium forms a system of branching ducts connected to the nipples and lobules where the milk is produced. Other parts of the breast provide a substrate for glandular epithelium development and functions [45]. The neovascular and lymphatic structures of the breast provide nutrition for breast cells and drain fluid into lymph nodes, respectively [46]. Anatomically, two-thirds of the breast is placed anterior to the pectoralis muscle with the rest elongating from the margin of sternum out to the midaxillary line. The tail of breast tissue known as the ‘axillary tail of spence’, is prolonged into the axilla [45]. The breast contains various cells including epithelial cells, fibroblasts, and immune cells. Epithelial cells grow from the nipple into a fat pad, formed by adipocytes, and infiltrated by vascular endothelial cells [47].

Insertion of an implant into the breast cavity depends on various factors such as the patient ‘s breast size and anatomy. An incision (infra-mammary, peri-areolar, trans-axillary, or trans-umbilical) is made to place the implant either in the sub-glandular (Fig. 2a) or dual-plane/ partial under the muscle or sub-pectoral [48] (Fig. 2b). If the patient has enough natural breast tissue the sub-glandular placement is the obvious choice for surgeons as it is easier for surgery and has better outcomes in terms of patient satisfaction [48]. However, placement of the implant in the sub-glandular position is more palpable and studies have indicated that the risk of capsular contracture is higher [49].

### Implant-host tissue dynamics following implantation

**Fig. 3:**
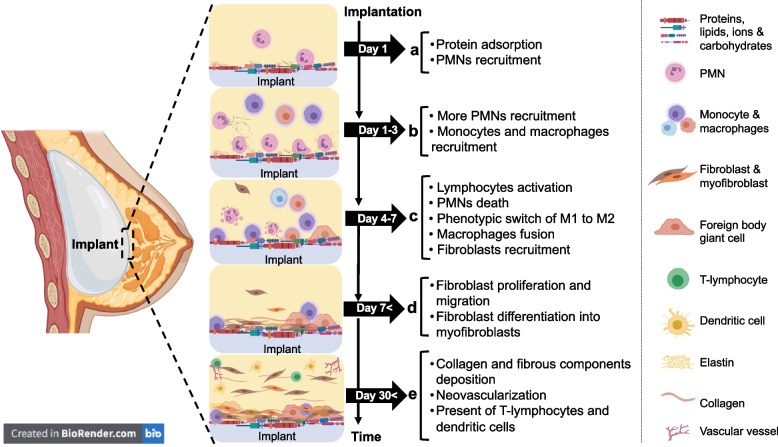
Sequence of events associated with host response following breast implantation

Following surgical incision and implantation, the local release of chemical mediators by injured cells results in a wound healing process characterised by an early inflammatory response and subsequent wound healing events [41, 42] (Fig. 3). Vasoactive substances cause changes in blood flow and vascular permeability leading to extracellular fluid and cells moving from the circulatory system to the injured tissue in a process called exudation [41, 42]. Both the extracellular fluid and migrating cells interact dynamically with the implant surface (Fig. 3).

#### Protein adsorption and provisional matrix formation

Immediately after implantation, mixtures of proteins, lipids, ions, and carbohydrates from the blood and wound bed competitively adsorb onto the implant’s surface [50–52]. This mixture may desorb, displace, or change conformation as well as denature until an enriched layer of stable proteins is formed [53, 54]. The rapid exchange of proteins on the implants’ surfaces was first described by Vroman and Adams in 1969, and it is commonly mentioned as a Vroman effect [55, 56]. Concentration, rate of diffusion, and affinity of proteins are influential in the adsorption of proteins on the implant surface. Small proteins found in a high concentration such as albumin, tend to adsorb on the implant first, but are gradually replaced by larger and higher affinity proteins such as fibrinogen and collagen [57, 58].

Various mechanisms have been proposed to describe the exchange of proteins adsorbed on solid surfaces and these include i) adsorption/desorption, ii) competitive exchange, and iii) exchange via transient complex formation [59, 60]. *In vivo,* however, protein adsorption is a highly complex process and not yet wholly understood as a vast number of proteins are involved. Importantly, various proteins are continuously secreted by cells including the ECM proteins. For instance, in a proteomic study of adsorbed proteins, Barr and colleagues identified 822 proteins on silicone implants’ surfaces after breast implantation [61]^.^ Furthermore, the affinity of proteins to the implant surface is altered by various other parameters, including the physio-chemical properties of the implant’s surface and other proteins’ presence on the surface.

**Fig. 4:**
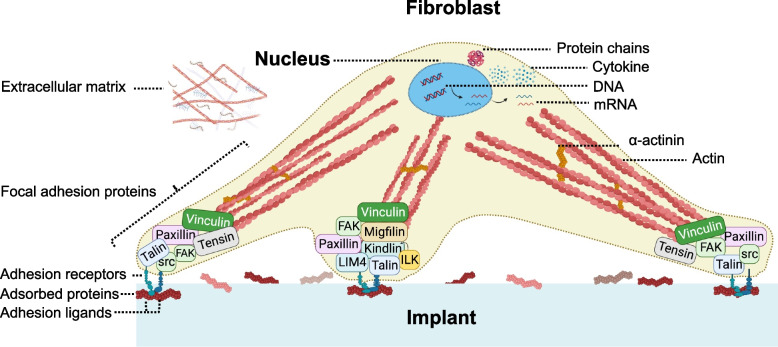
Schematic illustration of the cell-implant interactions via adhesion-mediating proteins and receptors. During attachment and spreading on the implant, the cell adheres to proteins adsorbed on the implant surface including collagen and fibronectin through transmembrane integrins units. Focal complexes are formed termed focal adhesion complexes consisting of molecular components such as paxillin, talin and vinculin

Local and recruited cells at the implantation site are much larger than proteins and thus move much more slowly. Cells encounter a dynamic layer of proteins covering the implant’s surface allowing attachment to occur. Some of these proteins, like von Willebrand’s factor, fibrinogen, vitronectin, and fibronectin, have specific peptide segments (e.g., arginine–glycine–asparagine (RGD) and proline-histidine-serine-arginine-asparagine (PHSRN) sequence) recognised by adhesion receptors on various cells. These cell recognition sequences facilitate cell attachment and subsequent tissue regeneration [62, 63]. Cell-surface interactions involving ECM proteins regulate cell behaviour through interactions with cell-surface receptors, such as integrin, and this results in tissue-specific cell spreading, migration, tissue assembly, and differentiation, as well as cell-to-cell communications (Fig. 4) [64]. Proteins prone to denature may induce the innate immune system whilst proteins in their native conformation do not [65].

One large protein known to bind to the implant surface is fibrinogen. Fibrinogen has a large size of 330 kDa and is one of the most abundant proteins involved in promoting adhesion of platelets, neutrophils as well as macrophages bonding to each other via αIIbβ3 (cluster of differentiation 41 (CD41)/CD61, GPIIb/IIIa), α_M_β_2_ (CD11b/CD18, Mac‐1, and Cr3), and α_X_β_2_ (CD11c/CD18, p150,95) integrin [66, 67]. Additionally, fibrinogen plays a crucial role in the blood clotting process by forming a fibrin network through the coagulation cascade [68].

As a result of protein-implant interactions, a provisional matrix with three-dimensional structural integrity develops at the tissue-implant interface, consisting of fibrin, inflammatory mediators, trapping cells, proteins, and platelets. This matrix is the initial ‘blood clot’ or ‘thrombus’ from the wound healing process formed through the conversion of fibrinogen to fibrin which is mediated by the initiator of activated factor XII/ Hageman factor (intrinsic pathway) or factor III/ tissue factor (extrinsic pathway) [41, 42, 69]. Macrophage-derived interleukin-10 (IL-10) has been shown to be involved in the cleavage of fibrinogen into fibrin, the major component of the provisional matrix [15]. Production of interleukin-10 by macrophages occurs concurrently with inhibition of tumour necrosis factor-alpha production [15]. Simultaneously, platelets secrete platelet factor 4 (PF4), platelet-derived growth factor (PDGF), and transforming growth factor beta (TGF-β), which play a part in leukocyte, fibroblast, and platelet recruitment [70].

To summarise, protein adsorption on the implant’s surface is necessary for wound healing. However, from the cell and histocompatibility perspective, if the thickness of the protein layer increases or specific protein conformation enhances cellular adhesion, this can lead to excessive capsule formation.

#### Inflammation

Injury to the skin and breast tissue followed by insertion of the implant induces FBR and sets in motion a series of events known as inflammation. Inflammation allows the body to clean up any cellular debris and infectious organisms and initiate repair [42]. The first stage of inflammation referred to as acute inflammation begins with the infiltration of polymorphonuclear leukocytes (PMNs) such as neutrophils, and mast cells to the injured tissue in response to diverse endogenous damage-associated molecular patterns (DAMPs) released from necrotic cell death [71].

Chemo-attractants produced by activated complement products, platelets, endothelial cells, and bacteria during implant insertion induce inflammation [72–75]. In turn, PMNs and later monocytes migrate to the injured tissue to phagocytose invading pathogens and cellular debris. Adsorption of ECM proteins such as fibronectin on the surface of the implant can increase neutrophil extracellular traps (NET) that are involved in elimination of infection agents through the action of proteases attached to chromatin filaments [76]. Over time, neutrophils are depleted via apoptosis either spontaneously or via the action of macrophages (efferocytosis process) [77, 78]. Breast implants cannot be engulfed by phagocytic cells due to their large size (> 10 µm) and this leads to secretion of mediators of degradation, such as reactive oxygen intermediates, degradative enzymes, and acids that may damage the implant and the surrounding healthy tissue [79–81].

The implant surface properties, type of adsorbed proteins, and the extent and duration of inflammatory responses may lead to chronic inflammation. In addition, infection and movement of the implant within the breast pocket (tribology) may additionally prolong the process of acute inflammation leading to chronic inflammation [82, 83]. Single macrophages fuse to form the multi-nucleated giant cells, known as foreign body giant cells (FBGCs) that attempt to phagocytose the implant. The inability to engulf implants [84], causes FBGCs to change to a “frustrated phagocytosis” state and remain on the implant’s surface via podosome adhesion (not focal contacts) [85]. Several inflammatory associated cytokines including IL-4 and IL-13 produced by immune cells at the injury site cause macrophage fusion [86–88]. Furthermore, chemo-attractants such as the chemokine (C–C motif) ligand 2 (CCL2) are also involved in directing macrophages towards each other [89].

Various pieces of evidence show that the von Willebrand factor, which was already adsorbed onto the surface, can reduce macrophages’ adhesion to the implants, leading to a decrease in FBGCs formation. In contrast, Immunoglobulin G (IgG) adsorption on the implant surface strongly provokes macrophage adhesion in the long term [90]. McNally and colleagues [87] demonstrated that vitronectin, interacting with integrin adhesion receptors of integrin alpha V beta-1 (αVβ1) and integrin alpha M beta-2 (αMβ2), can considerably promote macrophage adhesion and IL-4-induced FBGC formation. It has been demonstrated that the involvement and interaction of specific cell types, adsorbed proteins, cytokines, and chemokines can lead to induction of both acute and chronic inflammation at the injury site after implant insertion [87].

#### Fibrotic encapsulation

Adherent FBGCs/ macrophages and accumulation of collagen from fibroblast causes the persistence of FBR. Several reports indicate that when cells attach to the implant and release several cytokines, this influences FBR and induces signalling transduction [18, 91]. Many of the induced signalling cascades are involved in stimulating ECM synthesis and release of cytokines and growth factors [18, 82, 91, 92]. Fibroblasts are known to occupy most spaces on the material/ implant surface where they deposit several ECM proteins such as type I collagen [93–95]. ECM proteins deposited on traditional smooth implant surfaces are aligned nicely, whilst those deposited on rough implant surfaces may not be optimally oriented [96, 97]. The alignment of ECM proteins such as collagens, facilitates implant isolation in a fibrous capsule [98]. In addition, if apoptosis of granulation tissue does not occur, fibrotic encapsulation of the implant occurs [99].

**Fig. 5:**
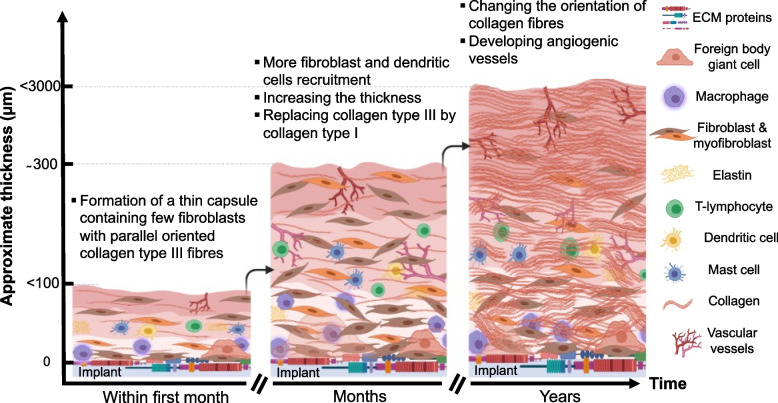
The development of a capsule over time after breast implant insertion. Days after insertion of the implant, cells such as granulocytes, lymphocytes, and fibroblasts surround the implant and contribute towards the formation of a normal and thin capsule. Over time, more fibroblasts and dendritic cells can be recruited to the implant site, leading to synthesis of more ECM proteins. Type III collagen in the initial provisional matrix is replaced with type I collagen as more fibrous ECM is formed around the implant. Encapsulation is part of the normal physiologic FBR, however, excessive deposition of fibrous ECM around the implants contracts the implant and cause capsular contracture

Dolores and colleagues demonstrated that the fibrous capsule removed from patients at various times after silicone breast implant surgery is mainly composed of three layers; (i) a layer touching the silicone implant, mainly composed of macrophages and fibroblasts (ii) an intermediate layer of connective tissue with internal vascular vessels, and (iii) an outer layer of dense connective tissue with external vascular vessels [90]. Moreover, T-lymphocytes CD4 + and dendritic cells (CD1a/CD208 + cells) are present in the inner layer in contact with the silicone implant and contribute toward immune response in the fibrous capsule. A high amount of fibronectin and tenascin were also detected in the first layer that mediates cellular and ECM protein interactions [90]. Within days of implant insertion, granulocytes, lymphocytes, and fibroblasts surround the implant and contribute towards the formation of a provisional matrix (Fig. 5). Over time, more fibroblasts and dendritic cells are also recruited to the implant site. Type III collagen in the initial provisional matrix is replaced with type I collagen as a fibrous capsule form around the implant (Fig. 5) [100–103].

Whilst encapsulation is part of the physiologic FBR, excessive deposition of fibrous material around the implants contracts the implant and leads to progressive capsular contracture. Silicone implant-related capsular contracture is associated with pain, discomfort, and hardness, and deformation of breast tissue. The amount of capsule around the implant varies and is linked to implant features such as surface topography and wettability. A histological analysis by Glicksman and colleagues demonstrated that textured implants as well as the continued presence of shearing forces are linked to enhanced development of FBR leading to the formation of double capsules [104]. Various studies have shown that implants of different roughness and hydrophobicity influence implant biocompatibility/integration and may play a key role in the development of complications post-implant augmentation [15, 105, 106].

### Silicone breast implant-related complications

Silicone breast implants have received both conflicting reviews since their first use decades ago. Despite the great benefit derived from silicone implants, their use and the resulting issues have led to increased scrutiny [107]. Questions about the safety of silicone breast implants arose following concerning reports on the possible link between certain types of textured silicone breast implants and specific neoplastic changes. Definitive studies showing the determining factors for someone to develop these complications are still lacking. Textured implants were introduced to increase the integration of the implants in the human body [1, 108]. However, textured implants were significantly associated with increased of development of anaplastic large cell lymphoma [109, 110]. Furthermore, other adverse effects include capsular contracture formation, implant leakage/rupture, site-specific complications and silicone particulate migration to different body tissues including the lung and skin [111]. Particulate debris and gel leakage can result in migration of silicone to other parts of the body which can also interfere with the detection of cancer. To overcome some of these problems, new and novel silicone implants are being introduced including the introduction of the Motiva SmoothSilk® / SilkSurface®. Some of these new implants have more than one layer of silicone in addition to a blue seal layer which prevents silicone gel leakage through the shell [112].

#### Implant-related capsular contracture

Breast augmentation has been associated with several complications and these include bacterial infection, scarring, hematoma, capsular contraction, and a rare cancer known as breast implant associated ALCL [109, 110]. Of these, capsular contracture is the most common complication associated with breast augmentation and in most cases requires surgical correction [109, 110]. Several reports indicate that thousands of implants are either removed or replaced each year globally [113]. Although estimates differ, a minimum of a fifth of breast augmentations will require corrections within the first 10 years after placement of the implant [114]. Capsular contracture is the main reason for surgical corrections post-breast implant augmentation [39], as the risk of developing and recurrence of capsular contracture continues to be relatively high [115, 116]. Ideally, the protective capsule formed must be a thin layer of fibrous proteins. However, a persistent fibrotic process can lead to a thicker capsule which hardens into the pathogenic capsular contracture [98, 117]. Bacterial infection (mostly opportunistic during surgery) can result in persistent presence of inflammatory cells around the inserted implant [118]. The resulting inflammation cause the body to synthesize ECM molecules in a bid to isolate the affected area [119]. Chronic inflammation can lead to excessive ECM synthesis and formation of capsular contracture [120]. Furthermore, bacteria can easily attach to adsorbed proteins on the implant surface via adhesins [121]. Whilst the early stages of capsule formation do not show any physical distortions to the breast, the continual tightening associated with capsular contracture eventually results in complications including malposition and discomfort for the patient [116, 122]. In this scenario, a regular check-up and replacement of the implant is recommended to detect the onset and treatment of capsular contracture formation [123, 124]. Implants of different textures and roughness have been associated with varying degrees and severity of capsular contractures [125, 126].

A clinical grading system approved by the Food and Drug Administration, referred to as the Baker grading system, is commonly used to describe the extent or degree of capsular contracture [127]. The Baker grading system groups the capsular contracture into four grades, with grade I used for breasts with a natural feel which physically looks normal [127, 128]. In this grade both the capsule and breast are normal. Grade II is when there is minimal capsular contracture and the breast is slightly firm when touched [127]. Both grade III and grade IV display symptoms. Grade III is characterised by moderate contracture with the breast feeling hard to touch whilst grade IV is used for severe contracture which is felt by the patient as well as displaying physical deformities seen by the doctor [127]. Reports show that the development of new and improved implants has resulted in decreased cases of capsular contracture [129–131]. Several reports indicate that certain types of texturisation of implant surfaces may have played a role in reducing the rate of capsular contracture formation [130, 131]. Randomised clinical trials, however, have shown that certain textured implants were associated with lower cases of capsular contracture compared to traditional smooth surface implants [132, 133]. Controlled clinical trials involving many patients demonstrated that patients receiving the traditional smooth surface implants are likely to develop capsular contracture compared to those receiving certain textured implants within 5 years [134, 135].

Conflicting reports are available with other reports suggesting that the placement position of textured implants matter. For example, the placement of implant under the glandular tissue is associated with capsular contracture whilst placement of implant under the pectoral muscle do not show any link to development of capsular contracture [136, 137]. In addition, improvements in surgical techniques and modifications that decreased biofilm development have helped decrease cases of capsular contracture [49]. Furthermore, prevention of gel bleeds as well as rupture of the implants, also aided in decreasing cases of capsular contracture [138, 139]. One of the issues associated with many clinical studies is the lack of long-term outcomes as well as providing adequate follow-up details about the study itself [140, 141].

#### Implant-related anaplastic large cell lymphoma

About a decade ago, the Food and Drug Administration reported a possible association between a cancer of the breast called ALCL and the use of textured breast implants [142, 143]. ALCL is a rare T-cell non-Hodgkin lymphoma that occurs within 10 years of implant insertion [143, 144]. Although ALCL was first described in 1997, the World Health Organization (WHO) only recognised it as a cancer of the breast in 2016 [113]. Most of the cases reported so far occur in countries with high usage of breast implants including the USA and Australia [145, 146]. Although the number of cases of ALCL is low, this may be due to under-reporting.

ALCL is characterised as a persistent fluid with cancer cells or a mass of cancer cells attached to the fibrous capsule and mostly occurs in one breast [147]. In some cases, ALCL can spread to lymph nodes. Reports show that ALCL is linked closely to the use of textured breast implants with high surface areas [146, 148–150]. Various theories have been presented on the origin of ALCL, from bacterial contamination to extreme inflammation linked FBR. ALCL can cause pain and discomfort to patients. Most reported studies linking ALCL to bacterial inflammation are based on the association [151–153]. Importantly, many studies have suggested a multifactorial cause for the development of ALCL ranging from bacterial infection [154], implant surface texture [155, 156], genetic factors [157], and mechanical friction [158]. Removal of the neoplastic and fibrotic tissue, as well as the implant itself are some of the ways to effectively treat ALCL [149]. Chemotherapy may be given to patients with advanced disease as well as those with a possibility of metastasis [159, 160].

Cancerous cells found within the ALCL seroma display horseshoe-shaped nuclei in addition to being anaplastic lymphoma kinase negative [161]. These cells are also CD30-positive and express T-cell markers including CD3 and CD4. Other markers expressed by these cancerous cells include CD25, CD80, and Interleukin 6 (IL-6) [162–164]. It is suggested that inflammation can cause T-cells to proliferate excessively [163] and together with cytokines involved in lymphocyte division, this may lead to some cells becoming tumorigenic. This tumorigenic process is then driven further by cytokines such as IL-6. Together with TGF-β and IL-10, IL-6 is thought to induce immune suppression, further promoting tumorigenesis by inhibiting host anti-tumour immunity [163, 165]. Another unproven theory is the association with development of biofilm infection in addition to a person’s genetic background that may trigger cell proliferation, which is acted upon by selection pressure to yield proliferation of a cell with proliferative advantage, leading to ALCL [165].

Several studies have supported the theory that biofilm infection can cause ALCL [150, 157, 166]. Silicone implants’ hydrophobicity can cause non-specific adsorption of various blood and wound fluid proteins leading to fouling. This in turn promotes growth of several bacterial strains on the implant surface [167]. For example, it was shown that *Helicobacter pylori* infection can cause inflammation over time, which is now recognised as a causal agent in the development of lymphoma of the gut [168]. *Staphylococcus epidermis* contamination on the implant surface can cause enhanced fibrosis [169]. A recent study reported high levels of *Ralstonia picketti* in ALCL-linked breast implants [151]. In addition, antibiotics are known to cause remission of the disease in many patients. Doxycycline treatment of *Chlamydophila psittaci* causes regression of adnexal marginal zone lymphoma [170]. Several studies have shown that textured implants may promote bacterial growth more than smooth surface implants. Bacterial growth on textured implants can cause inflammation, leading to tumorigenesis and the development of T-cell lymphoma [166, 171, 172]. More lymphocytes have been observed to attach to textured implants versus smooth surface implants and a positive link has been found between bacterial growth and lymphocytes attached to implants [166, 171, 172]. Most importantly, biofilm has been detected on clinical samples of ALCL via quantitative polymerase chain reaction (qPCR) as well as visual analysis using scanning electron microscopy and fluorescent *in situ* hybridisation [151]. A large proportion of the bacteria in ALCL were identified by sequencing studies as Gram-negative [151].

The described complications associated with silicone breast implants suggest that the implant properties (physical and chemical) impact on biocompatibility. An optimised silicone surface via modifications would enhance the implant biocompatibility and reduce the occurrence of complications.

## Silicone implant properties

**Fig. 6:**
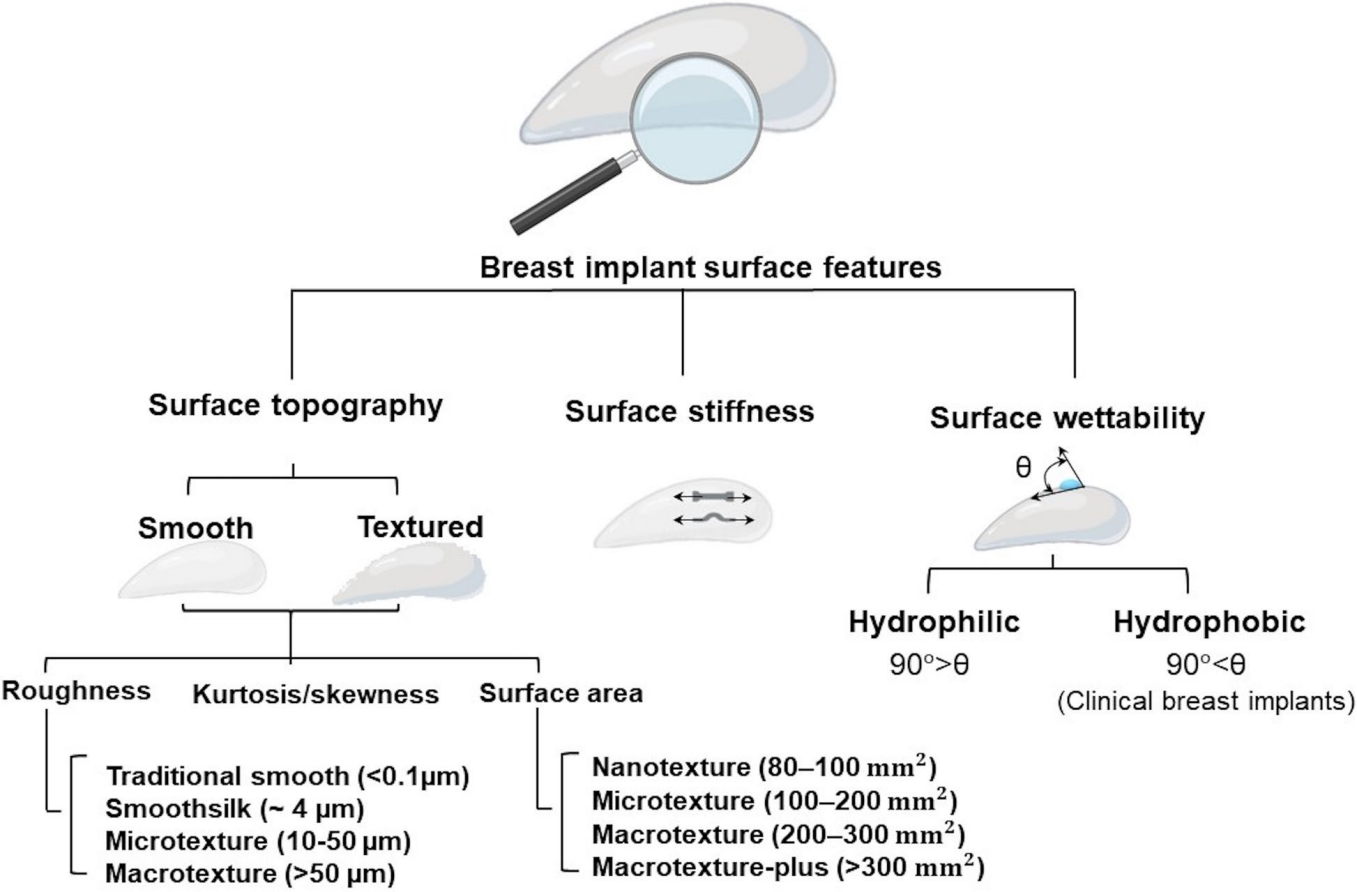
Silicone implant surface properties influencing cell-implant interactions include surface topography, stiffness, and wettability. Topography can further be divided into surface roughness, kurtosis and surface area

The implant’s physio-chemical properties mainly influence the type and number of proteins adsorbed onto its surface and the subsequent inflammatory cell adhesion and FBR. Importantly, silicone surface implant properties can determine the fate of the breast implant in the body (Fig. 6). To ensure that silicone breast implants have optimal biocompatibility and display a desirable biological response, researchers have investigated strategies that minimise FBR by focusing on improving shell and gel properties. This review discusses recent modifications and advances in implant surface properties aimed at improving cell adhesion and enhanced implant integration in the body.

### Surface topography

Implant’s surface topography, including profile shape and roughness influence the types of proteins that adsorb onto the implant surface as well as their conformation and ultimately impact cell orientation alongside the implant [173]. In addition, the orientation of collagen fibres deposited around the implant is impacted by the implant surface topography [136, 174].

Extensive research has been performed on the surface topography of breast implants aiming to control cellular response and ultimately influence implant fate in the body. For example, coating fine-cell polyurethane, a technique invented by William J Pangman [175], on the implants’ surface, formed open-pore texture leading to better tissue ingrowth and supposedly low tendency to capsular contracture [176]. However, polyurethane-foam-covered silicone implants gradually degraded *in-vivo*, producing toxic hydrolysis material, and caused delayed FBR [177, 178]. These silicone breast implants were therefore withdrawn from the United States of America implant market [179]. Soon after, modifications of silicone surfaces resulted in surfaces showing better biointegration and reduced FBR with each surface having a distinct microenvironment influencing cell shape and, thus, bio-integration depending on their texturising method [22, 179]. Various methods are used to achieve texturisation and these include imprinting, salt-loss, and gas expansion technique. Whilst the main aim of texturisation is to prevent implant malposition as well as easy maintenance, biointegration of silicone implants remains an issue requiring further investigations.

It has been shown that collagen fibrils around textured breast implants have multiplane conformation that causes more flexible and thinner capsules and are less likely to cause contraction [180]. In comparison, collagen fibres are organised longitudinally and parallel to the traditional smooth implants during early inflammation, and gradually change their orientations in the long term [181, 182]. Capsule formation is enhanced when fibres are aligned longitudinally. Contrasting data on histologic tissue responses and clinical outcomes on textured shells demonstrated the superiority of textured surfaces over smooth surfaces in decreasing capsular contracture and reoperation [125, 126, 136, 183–186]. For example, Poeppl and colleagues demonstrated that patients with textured breast implants showed reduced degree of symptoms for capsular contracture than patients who had smooth-surfaced implants [125]. Fischer and co-workers showed that textured silicone implants resulted in temporarily thicker but overall less dense fibrotic capsules compared with smooth surfaces in an animal model [187]. Moreover, Bergman and co-workers showed that textured implants can enhance bacterial contamination leading to increased risk of capsular contracture [188].

Properties of textured implant surfaces including size and shape influence soft tissue adhesion. Deep pores (> 350 µm) have demonstrated increased fibrous capsule disruption and enhanced tissue ingrowth into textures than smaller pores, leading to decreasing the risk of capsular contracture and implant movement respectively [22, 181]. Furthermore, textured implant surfaces, increased surface area for attachment compared to smooth surfaces, allowed tissue ingrowth and more adherence than smooth surfaces [22]. Tolksdorf and co-workers [189] recently investigated the effect of different pore sizes of silicone textured surfaces produced by the salt-loss technique on human fibroblast behaviour in an *in vitro* model. The authors’ results showed that textured surfaces with fine (< 65 µm) and medium (> 250 µm) pore size leads to a compact uniform cell layer with good adhesion. In contrast, the fibroblasts could not bridge the coarse texture with larger pores (> 500 µm), causing irregular growth and finger-shaped cell extension missing the intracellular contact. Moreover, the expression of TGFβ1, a key molecule for differentiation of fibroblast to myofibroblast and creation of capsular contracture, was lower on fine and medium texture compared with coarse ones [189].

Topographical features may provide areas that are desirable for focal adhesion formation [190]. A one-week examination of fibroblast cytoskeleton orientation on different smooth and microtextured surfaces has shown that microfilaments and the vinculin-containing attachment aggregations could orient toward several directions on the surfaces with large ridges (5 and 10 µm). In contrast, on surfaces with the small size of ridges (2 µm), they attached only in the direction of ridges [190].

To understand the effect of surface topography on cell behaviour, it is important to consider the topographical aspect ratio (depth/width) instead of one feature dimension (width, depth, or pitch). The increase in the aspect ratio of textures on the surface increases cell alignment and elongation regardless of material and cell type [191]. Depending on the texturising method, the shapes of textures are different [28, 192]. The cross-section and surface topographical examination of 17 types of breast implants have shown they typically fit into three categories; i) regular peak and valley structures with heights of low amplitude (PV-patterned surfaces) ii) curve-shaped open cavities features of amplitude ranging from 50 µm to 300 µm (OC-patterned surfaces), iii) repetitive and regularly distributed unsealed cuboid-like patterns of high amplitude (> 400 μm) (semi-opened cavities (SOC)-patterned surfaces) [28]. The respective cross-section of these categories is given in Table 2. Overhanging features of some cuboid structures can help fix the position of ingrowing tissue and device [17]. The coated-salt-loss technique or salt-loss technique followed by brushing can generate this structure [28, 193].

**Table 2.**
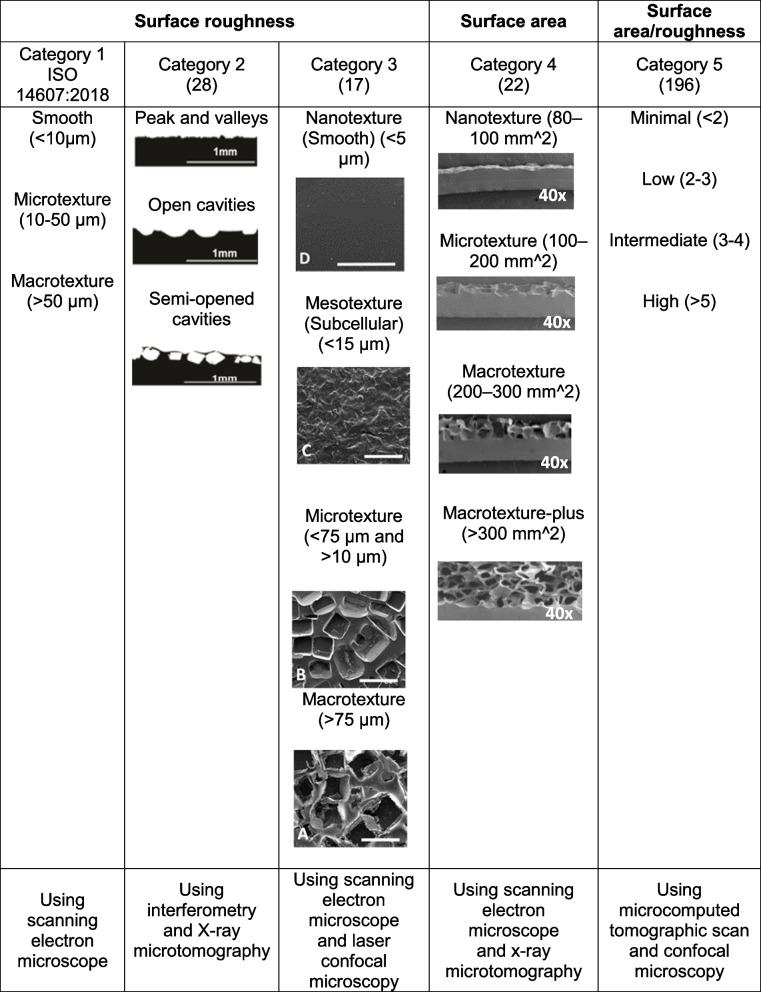
Surface topographical categories

An animal study of capsule formation and tissue adherence of different breast implant surfaces at six weeks after implantation revealed that surfaces with larger surface area textures develop unstructured alignment of collagen fibrils at their tissue-implant interface and need stronger force for detachment of the implant tissue [22].

Another important parameter to characterise surface topography is surface roughness. The surface is considered rough if the actual surface’s vertical deviations against its ideal form are large and smooth if the deviations are small [194]. Surface roughness parameters include height descriptors (e.g., arithmetic mean height (Ra), surface skewness (Ssk), and kurtosis value (Sku)) can manipulate friction and cell adhesion and eventually tissue response. Surface roughness is associated with increased surface area [14], and contact angle (hydrophobicity) [195], but this depends on the nature of the material analysed. Increased implant surface area is linked to enhanced cell adhesion, in particular adhesion of fibroblasts [19, 196]. Prasad et al. [197] showed that in the sub-micron scale (88–650 nm), an increase in the roughness of silicone elastomer substrates decreases fibroblast growth. Rough surfaces also induce macrophage (M1) activation to (M2) by up-regulating macrophage inflammatory proteins 1a (MIP-1a) and monocyte chemoattractant protein-1 (MCP-1) and downregulating the secretion of chemokine C-X-C motif chemokine ligand 10 (CXCL10) [198]. Surface topography plays a key role in directing extracellular matrix related gene expression [28]. A study involving the use of breast implants with semi-opened cavities-patterned surfaces demonstrated that metalloproteinase inhibitor 4 (TIMP4) and tumour necrosis factor ligand superfamily member 11 (TNSFS11) expression were remarkably down-regulated. In addition, matrix metallopeptidase 9 (MMP9), matrix MMP12, TIMP1, and IL-8 were drastically up-regulated compared to other surfaces with peak and valleys and open cavities patterned surfaces [28]. Overall, evidence shows that the progression of FBR is dependent on many factors including cytokines and MMPs. These soluble factors are secreted by various cells including fibroblasts, macrophages, and inflammatory cells. Chemokines and MMPs cause a series of cellular events associated with FBR such as invasion, blood vessel formation, migration and fibrosis. Constant changes that occur during FBR means that the amounts and type of soluble mediators available differs at various time points.

The International Organization for Standardization (ISO) in ISO 14607:2018 [199] has defined three categories based on the value of average surface roughness; smooth: < 10 μm; microtexture: 10–50 μm, and macrotexture: > 50 μm and given guidance on the assessment of the physical surface characteristics of silicone implants. Many scientists within the materials field have suggested alternative classifications of texturisation. As a result, many studies have used surface topography parameters including roughness [17, 28], and surface area [22, 192] in order to classify textured breast implants (Table 2).

Despite displaying several advantages, studies on textured implants report long-term complications such as late seroma, double capsule, and anaplastic large cell lymphoma (ALCL) [200–203]. As a result, new breast implants with smooth surfaces have been introduced and shown fewer complications [112, 204]. The three-dimensional (3D) imprinting technology is used for these novel implants to create low roughness features and micro/nano topographical structures with high contact points on PDMS [112]. Since the surface topographic properties of these new implants are less than the cellular level, tissue ingrowth is impossible [17]. Furthermore, the surface’s high contact points prevent fibroblast clumping, presumably decreasing capsular contracture risk [112]. In addition, immunophenotypic characterisation has shown that on these surfaces, the secretion of tumour necrosis factor α (TNF-α) and IL-1β (inflammatory cytokines) by cells is lower than when cells are cultured on other surfaces [23].

### Surface stiffness

While more studies have focused on implant surface topography, the formation of implant-cell adhesion complexes rely on the softness and stiffness of the implant’s shell [205]. The stiffness of substrates is influenced by thickness and can be calculated through measuring the elastic modulus [19]. The elastic modulus (E) of various commercial breast implant shells was measured and shown to be around 600–3000 kPa, values higher than the elasticity of the human dermis (E < 10 kPa) and human mammary tissue (E < 0.5 kPa) [19, 205, 206]. Cells have been shown to spread more on substrates with high stiffness, with F-actin cytoskeleton networks being more organised. In contrast, cells assume a round shape with restricted F-actin complexes on soft substrates [207–209]. In addition, stiffness influences ECM protein secretions, thus, regulating ECM ligand depositions for cell attachment [210].

An *in vitro* assessment of neutrophil activities on silicone surfaces with different stiffness (0.2–32 kPa) revealed that stiffer surfaces evoke NET formation and more pro-inflammatory cytokines secretion [76]. Noskovicova et al. [205] recently investigated the effect of breast implant surfaces with different stiffness on development of fibrosis capsule in a mouse model and showed that a softer silicone layer (E ~ 2 kPa) on the breast implant reduce collagen deposition and myofibroblast activation. The reduced implant fibrosis was a result of less intracellular stress, decreased recruitment of αv and β1 integrins, and TGF-β1 signalling [205]. The same study showed that stiffness does not affect the numbers of macrophages and their polarization states *in vitro* [205].

### Surface wettability

In terms of appropriate body response to implant and biocompatibility, wettability is a critical property of the surface. Wettability is defined as the capacity of a liquid solution to remain in contact with solid surfaces and is quantified through contact angle measurements [211]. Silicone devices have low wetting ability due to their chemical composition and are therefore considered hydrophobic. The helical structure of PDMS as well as the presence of protruding methyl groups results in a hydrophobic material. The contact angles of clinical breast implant surfaces have been measured between 108° and 142° which indicate all surfaces are hydrophobic [17, 19]. Textured implants depict 17–27% increase in contact angle and hydrophobicity compared the smooth surfaces [17, 19].

Wettability influence protein [212, 213], platelet [214], cell [215], and bacterial [216] adhesion. The more hydrophobic the surface is (shown by a contact angle > 90^°^), the more biomolecules such as proteins have an affinity to it [212, 213]. A surface with optimal wettability (not strongly hydrophilic or hydrophobic) adsorbs enough proteins with appropriate conformation and receptor-ligand accessibility to allow cell attachment [217]. The optimum contact angle of the surface for proper cell adhesion and cell growth is varied and depends on the kind of cell. For instance, A contact angle around 70° (moderately hydrophilic) is reported optimal for *in vitro* culturing fibroblasts [218], as the most abundant cell type in the breast tissue [219]. To improve wetting properties and biocompatibility of silicone implants, surface chemistry modifications of silicone have been the subject of many studies in recent decades [220–226].

## Silicone surface coating and modifications

Silicone (PDMS) with repeating (CH3)_2-_Si–O- units has a hydrophobic character due to the nonpolar methyl group presence in its chemical structure. In turn, high amounts of proteins become adsorbed on the silicone and strongly interact with its surface, which leads to protein denaturation and loss of their biological activity. As a result, cell detachment occurs over time and cells cannot bind to the denatured proteins on the implant surface [227]. Moreover, bacteria and inflammatory cells readily adsorb on the silicone surface, causing biofilm formation and extensive fibrous encapsulation [12].

Immobilising polar groups on the surface of silicone can improve wettability. However, providing a homogeneous stable grafting on the surface in order to regulate the implant surface’s wettability of silicone is challenging since the silicone hydrophobic chains easily migrate to the interface and recover the surface due to silicone’s low glass transition temperature (T_g_ ≈ -123 °C) [12, 228]. Therefore, in most cases, modifications require pre-treatment steps.

### Pre-treatments and silanization

Immobilising hydrophilic polymers on the surface requires activating the PDMS surface, which can be done using chemical oxidation, UV/ozone/γ-irradiation, plasma-induced techniques and laser treatment [220–224, 229]. Through these pre-treatment techniques, Si-CH_3_ groups will be oxidised to polar species such as Si–OH or Si-OOH groups for a limited period. Then the intended polymer can be grafted covalently to active sites provided. This paper briefly discusses pre-treatment techniques below.

The most common technique used to activate silicone surfaces is oxygen plasma treatment under vacuum. In this technique, reactive oxygen groups are introduced on the silicone surface resulting in a reactive silica layer with induced silanol groups [230]. The presence of the silanol groups enhances the silicone surface wettability for a limited time. To maintain activated silicone surface hydrophilicity for longer periods for example weeks, de-ionised water and Luria–Bertani broth can be used [106, 231]. Plasma activation has been shown to inhibit bacterial adhesion but has limitations as it can cause silicone surface cracking [232]. Importantly, oxygen plasma exposure can influence silicone surface roughness, as determined by atomic force microscopy. Another form of plasma activation is corona discharge. In this case, plasma treatment is localised and is performed under atmospheric pressure [233, 234]. Gas molecules are ionised and converted into radicals that react with the silicone surface. Corona plasma results in few molecules being oxidised but still adds polar groups to the silicone surface. Corona plasma treatment, similar to oxygen plasma, can also cause silicone surface cracking [235].

UV/ozone, via a mercury-vapor grid lamp, can be used to excite atoms on silicone surface. UV light at different wavelengths is used in a stepwise process to convert O2 to O3 and then into radicals or active species on the silicone surface. This results in the conversion of methyl groups into -OH groups [236, 237]. Further modifications can be performed on the activated surfaces with -OH groups to enhance surface wettability. A faster way to activate silicone surfaces is by using UV irradiation. In this process, the silicone material is irradiated with UV at 365 nm using a Lot Oriel mercury lamp in degassed water for about an hour [238]. Reports indicate that this technique can result in long lasting -OH groups on silicone surfaces as determined by FTIR analysis [106, 238]. The activation of silicone surface via UV irradiation is thought to involve the formation of -CH2 groups from the methyl group on silicone as well as -OH groups from water molecules. The -CH2 and -OH groups combine to form -CH2-OH on the silicone surface.

The use of ultra-short and short pulsed lasers for very short durations creates very high energy flux at a focal point and this causes plasma-mediated ablation of PDMS material. Due to the short duration of the pulse, minimum thermal breakdown is recorded. Both ultra-short and short lasers have been used in laser micromachining of PDMS surfaces [239, 240]. Precise control of PDMS laser treatment can result in a variety of micro- and nano-structures that mimic natural tissues [241]. The PDMS surface treated with lasers can then be modified further for example through immersion in various solutions of natural and synthetic polymers.

In addition to the use of energy sources for silicone surface activation, chemical oxidation, chemical vapor deposition, and sol–gel strategies can be used. Referred to as Piranha, chemical oxidation utilising a mixture of strong acids such as sulphuric acid and hydrogen peroxide has been used to activate silicone surfaces [242]. Care is needed during this activation process, as persistent exposure to the strong acids can lead to degradation of the silicone surface [238]. As reported by Yang and colleagues, cracks can appear on the PDMS surface due to over-exposure to strong acids [242]. A solution to this problem includes the use of less aggressive acids such as nitric acid [106, 238]. Wettability obtained via the use of chemical oxidation can last for weeks and sometimes months. When a silicone surface is heated, gas molecules can be transformed into solids under vacuum conditions, leading to the formation of a thin layer of material. This process is referred to as chemical vapor deposition and has been used to modify silicone surfaces with hexamethyldisilazane, for example [243]. One major disadvantage of chemical vapor deposition is the loss of hydrophilicity within minutes. This problem can be overcome by combining chemical vapor deposition and silanization [244]. Furthermore, the production of materials from other small molecules on the surface of silicone can result in a thin layer of ‘new’ material with properties influencing surface interactions with cells. This is what the sol–gel technique is based upon. For example, SiO2 can be generated on silicone surfaces via the reaction of tetraethoxysilane and water [245]. In situ formation of titanium oxide on silicone surfaces has been shown to enhance hydrophilicity over several months [246, 247].

Lastly, both micro- and nano-patterning have been used to introduce different patterns on silicone surfaces. To achieve nanopatterning, an electron beam lithography procedure is done. In the case of micro-patterning, an optical lithography procedure is performed. The flexibility of PDMS material allows textures to be introduced on its surface via the use of lithography technique [248, 249]. Using a silicone wafer as a foundation, a layer of photoresist is added on top, followed by the introduction of texture patterns by photolithography. Finally, etching will generate designs for PDMS moulding. A layer of PDMS is added on top of the patterned master, cured and finally removed from the master. Cells cultured on such patterned PDMS surfaces display characteristic shapes and morphologies [250–252]. A combination of photolithography and casting is used to produce nanopatterned PDMS. A layer of PDMS is made on polyester film mould, heated, and then peeled off to allow replicas to be made from the PDMS surface.

### Silicone surface coating materials

A typical strategy to modify silicone hydrophobicity involves forming a passivating surface layer using hydrophilic material such as polyethylene glycol derivatives [225, 226]. Selected materials investigated for grafting on the silicone surface are summarised according to their chemical families (Table 3). These polymers are resista

nt to protein adsorption by increasing the wettability of the surface but may not resist bacteria and cell adhesion [225, 253]. Some of these materials, including high-molecular-weight hyaluronic acid (FDA-approved) and phosphorylcholine (PC), are biomimetic materials that could improve the implant’s *in vivo* stability and inhibit capsular contracture [254–257].

**Table 3:** Selected materials used to functionalise silicone surfaces

Chemical family		Name	Type of study	Effect	Ref
**Synthetic materials**					
Polyacrylate		Poly (methacrylic acid) (PMAA)	*In vitro*	• Increasing hydrophilicity • Decreasing bacterial adhesion	[220, 258]
		Poly (hydroxyethyl methacrylate) (PHEMA)			
		Oligo (ethylene glycol) methyl ether methacrylate (OEGMA)			
Polyether		Poly (ethylene glycol) (PEG)/Oligo (ethylene glycol) (OEG)	*In vitro* and *in vivo*	• Rapid, reproducible grafting • Increasing hydrophilicity • Increasing cell adhesion • Reducing fibrinogen, immunoglobulin G, and platelet adsorption • No significant inhibition of leukocyte adhesion • Reducing the thickness of the fibrous capsule	[225, 259–261]
		Methoxy(polyethyleneoxy) propyltrimethoxysilane (PEG-silane)	*In vitro*	• Reducing platelets adsorption • Stable grafting	[226]
Polyacrylamide		Poly(acrylamide) (PAAm)	*In vitro* and *in vivo*	• Increasing hydrophilicity • Decreasing fibroblast adhesion • No significant inhibition of leukocyte adhesion • Reducing the thickness of the fibrous capsule	[259]
Polyvinyl		Polyvinylpyrrolidone (PVP)			
		Polyvinyl alcohol (PVA)	*In vitro*	• Good neural response • Long-term stable hydrophilic surfaces • Improving cell growth and proliferation	[262–264]
Polystyrene		Poly (Sodium Styrene Sulfonate) (polyNaSS)	Material characterisation only	• Increasing hydrophilicity	[238]
**Natural materials**					
Zwitterionic	Phospholipids	Phosphorylcholine	*In vitro*	• Increasing hydrophilicity • Reducing the thickness of the fibrous capsule	[255]
		Poly (2-methacryloyloxyethyl phosphorylcholine) (PMPC)	*In vitro* and *in vivo*	• Increasing hydrophilicity • Significant reduction in inflammatory cell and cytokines recruitment • Reducing the thickness of the fibrous capsule	[265]
	Carboxybetaine	Poly carboxybetaine methacrylate) (pCBMA)	*In vitro*	• Increasing hydrophilicity • Reducing protein adsorption and cell adhesion	[266, 267]
	Sulfobetaine	Sulfobetaine silane (SBSi)	*In vitro*	• Increasing hydrophilicity • Stable grafting • Reducing protein adsorption • Decreasing bacterial adhesion	[226]
		Polyethylene glycol sulfobetaine silane (PEG-SBSi)			
		Polydopamine	*In vitro*	• Increasing hydrophilicity • Increasing cell adhesion	[268, 269]
Polypeptide		Collagen	*In vitro*	• Increasing hydrophilicity • Increasing cell adhesion • Unstable coating layer	[268]
		Silk fibroin	*In vitro*	• Increasing elasticity • Increasing cell viability	[270]
		Nε-myristoyl-lysine methyl ester (MKM)	*In vitro*	• High stability and long-lasting hydrophilicity in ambient and aqueous environments • Reducing fibrinogen adsorption • Decreasing bacterial adhesion	[257] [254]
		Poly-l-lysine (PLL)	*In vitro* and *in vivo*	• Inhibited capsular contracture	
Polysaccharides		Hyaluronic acid (HA)			
			*In vitro*	• Increasing hydrophilicity • Long-term stability • Reducing protein adsorption • Reducing bacteria adhesion	[271–273]
		Gelatin	*In vitro*	• Increasing hydrophilicity • Improving cell adhesion and growth	[253]
		Carboxymethyl cellulose (CMC)	*In vitro*	• Increasing hydrophilicity • Improve cell adhesion and cell migration • Reducing protein adsorption	[242]
		Carboxymethyl -1,3-dextran (CMD)		• Increasing hydrophilicity • Improve cell adhesion and cell migration • Reducing the adsorption of negatively charged proteins • Increasing the adsorption of positively charged proteins	
		Alginic acid (AA)		• Increasing hydrophilicity • Improving cell adhesion and cell migration • Reducing the adsorption of negatively charged proteins • Increasing the adsorption of positively charged proteins	

Various polymers have been utilised in coating procedures with the sole aim of enhancing silicone material biocompatibility. PDMS displays high hydrophobicity, and this negatively affect cell adhesion and growth and, in most cases, makes surface modification a necessity. Natural polymers including collagen, gelatine and hyaluronic acid are very good examples [25, 274, 275] whilst synthetic polymers include poly-lactic acid (PLA), poly-glycolic acid and poly-lactic-co-glycolic acid (PLGA) [276, 277]. Natural polymers have several advantages over synthetic polymers including having excellent biocompatibility and functional groups relevant for biological processes, they suffer from poor tensile strength. To overcome poor tensile strength, natural polymers can be combined with synthetic polymers that display good mechanical strength. Below, we briefly describe the use of various biopolymers in modifying the surface of silicone implants and the biocompatibility of the resulting surfaces.

#### Collagen

The connective tissue has an ECM mainly composed of collagens [278–280]. Although close to 28 proteins have been identified to belong to the collagen family, most of the collagens in the body are types I, II and III collagens [279]. Type I collagen is the most used collagen for coating silicone implants partly due to it being the dominant collagen within connective tissue and has been linked to enhanced fibroblast proliferation [281–283]. Being non-toxic as well as being biodegradable makes type I collagen a suitable material for coating silicone implant surfaces. Since collagen is biodegradable, over time the coating on the implant surface will be depleted resulting in bare silicone implant interacting with tissue. However, as the degradation of collagen happens over time, we speculate that biointegration would have occurred by then. A recent study by Sharma and colleagues showed that grafting collagen to silicone by means of polydopamine as a conjugator enhanced hydrophilicity resulting in better mesenchymal stem cell adhesion [268]. In another study, Li and co-workers showed that coating PDMS with collagen and polydopamine increased its biocompatibility enabling long culture of fibroblasts [284]. Gao and colleagues utilised L-3,4-dihydroxyphenylalanine to covalently immobilise collagen on the PDMS surface. The modified PDMS surface showed enhanced wettability and biocompatibility [285].

#### Hyaluronic acid

Also referred to as hyaluronan, hyaluronic acid is a viscous polysaccharide that has been utilised as a viscoelastic material in many processes [286]. Various reports have shown that hyaluronic acid is biocompatible, promotes cell adhesion and proliferation, processes that are important after implantation of silicone implants [287, 288]. Xue and colleagues demonstrated that modifying the surface of PDMS with hyaluronic acid conjugated to polydopamine increased surface compatibility and this allowed the resulting material to be used in medical implants [271]. Hung and colleagues demonstrated modifying the surface of PDMS with dopamine and hyaluronic acid increased its biocompatibility and allowed enhanced mesenchymal stem cell differentiation [272]. In another study, Yue and colleagues utilised both hyaluronic acid and collagen type I to functionalise the PDMS surface resulting in increased pheochromocytoma cell proliferation and differentiation [289]. In this work, the authors conjugated type I collagen onto the hyaluronic acid-modified PDMS leading to promotion of cell-substrate interaction [289]. Hyaluronic acid can be degraded by hyaluronidases and its long-term presence on silicone implant in the body is not guaranteed *in vivo* [290]*.* Studies investigating long-term *in vivo* stability of hyaluronic acid coating on silicone implants are not available.

#### Gelatin

Derived from collagen, gelatine is a biodegradable natural polymer that exists as type A and type B gelatine [291]. Several studies have shown that gelatine is biocompatible and promotes cellular adhesion to surfaces hence its use in tissue culture [253, 292]. Importantly, gelatine contains various functional groups that allow for cross-linking with other materials for many applications. Ai and colleagues investigated the crosslinking of gelatine with glutaraldehyde on silicone surface and revealed that this resulted in enhanced endothelial cell proliferation and adhesion [253]. Liu used proanthocyanidin-crosslinked gelatin on a silicone tube to investigate nerve fibre regeneration [293]. This study demonstrated that the proanthocyanidin-crosslinked gelatine on silicone surface promoted Schwann cell growth and promoted peripheral nerve regeneration [293]. Since gelatin is biodegradable its presence on the modified silicone breast implant in the body is not assured. Future studies need to investigate this concern.

#### Poly-glycolic acid and poly-lactic-co-glycolic acid

Poly-glycolic acid (PGA) has excellent strength and display great hydrophilicity as a polymer. However, its biggest disadvantage is that PGA can easily degrade causing a local increase in glycolic acid, a by-product of the degradation process. Elevated levels of glycolic acid result in local tissue damage which is undesirable if used on silicone breast implants. Hatton and Brook investigated the utility of reinforced silicone and biodegradable PGA in an *in vitro* study using a rat bone cell culture model [294]. The results demonstrated the osteoinductive properties of both silicone and PGA with bone cells showing increased proliferation and bone-like tissue formation within the weave of the PGA membrane [294]. When PGA is mixed with poly-lactic acid, poly-lactic-co-glycolic acid (PLGA) is formed. By controlling the amounts of its monomers, it is easy to control the hydrophilicity of PLGA. In most cases, PLGA is blended with other materials to develop products useful for tissue regeneration by encouraging cell proliferation. For example, combining PLGA and silicone was shown to enhance endothelial and smooth muscle cell proliferation *in vitro* [295].

#### Poly-vinyl alcohol (PVA)

A water-soluble polymer used in many applications, PVA has been shown to irreversibly bind to PDMS [296]. Trantidou and colleagues grafted PVA to the surface of plasma-treated PDMS and demonstrated that PVA deposition enhances PDMS surface hydrophilicity for increased time (above 30 days) [262]. By combining PVA coating and nano-patterning of PDMS together with exposure to air plasma, Li and colleagues demonstrated that the resulting hydrophilic PDMS surface was stable and promoted cell growth [263]. Using PDMS as an anchor, Huang and colleagues demonstrated that 3T3 fibroblasts on PVA-gelatin modified surface spread easily and had normal spindle shape than fibroblasts on PVA modified surface [297].

#### 2-hydroxyethylmethacrylate (HEMA), Oligo-ethylglycol-methylmethacrylate (OEGMA) and poly-ethylene glycol (PEG)

Most methods of chemical modification reported so far in order to improve wettability are laborious, and the final grafted surfaces are non-homogeneous which can affect the topography of silicone implants. Recently, Montoya-Villegas [220] introduced a method involving the use of reversible addition-fragmentation chain transfer (RAFT) to control graft distribution of HEMA and OEGMA on the surface of silicone rubber films oxidised with the γ-irradiation method. Their approach has potential in the modification of the wettability of silicone implants with control of grafting [220]. It has been shown that grafting hydrophilic groups increased the swelling of the modified PDMS leading to greater flexibility (low stiffness) in wet condition [220]. To improve the biocompatibility of silicone’s surface using PEG, Mikhail et al. [225] linked peptides and proteins to PEG and then introduced them to a pre-functionalised PDMS surface. Their method was reproducible, and the modified surface was durable [225].

#### Zwitterionic polymers

Zwitterionic materials with resistance to adhesion of bacteria and protein adsorption are the next generation of coating materials on silicone implants. They contain both positively and negatively charged groups, with water-binding capabilities, resulting in a highly hydrated surface [266]. Surface hydration is generally considered key to nonspecific protein adsorption resistance, as a tightly bound water layer forms a physical and energetic barrier to prevent protein adsorption to the surface [298, 299]. Moreover, zwitterionic polymers have strong salt bridge interactions, enhancing the coating’s stability [300]. Using 2-methacryloyloxyethyl phosphorylcholin, a zwitterionic PC-based polymer, Ham et al. [265] immobilised a durable coating on silicone breast implants which showed a significant decrease in the amount of inflammatory cytokines released by macrophages compare to uncoated implants. In addition*, in vivo* assessment of these coated implants showed a thinner, sparse capsule formation in a porcine model after six months implantation [265]. Hydrogel polymers are another possible option to improve the wettability of silicone using as a coating or composite with silicone rubber [264, 301]. Hydrogels entrap a high volume of water in their three-dimensional cross-linked network without dissolving in an aqueous solution. As a result, it has the potential to reduce foreign body reactions by decreasing protein adsorption on the surface [264].

## Summary and perspectives

Breast augmentation is the most common plastic surgical procedure performed to correct breast abnormality for both cosmetic and oncologic reasons. One of the common materials used in breast implants is silicone due to its inherent properties including ease of fabrication, oxygen permeability, flexibility as well as low cost. However, most macro-textured and traditional smooth surfaced commercially available breast implants made from silicone have demonstrated limited biocompatibility. Thus, it is of great value to develop strategies to modify the properties of silicone surfaces to enhance the bio-integration of silicone breast implants. Silicone properties such as topography, stiffness, and wettability influence cell adhesion to the implant and therefore bio-integration. In this review, we provide a critical synthesis of advances in silicone surface modification techniques and strategies to enhance silicone breast implants biocompatibility. The review starts by discussing the biological processes involved when the implant is inserted into the breast. This is followed by the presentation of literature on the normal and aberrant formation of a capsule around the inserted silicone implant. Silicone implant fundamental properties that influence the formation of the capsule include topography and wettability. Various methods have been developed to modify the silicone implant surface properties including wettability and topography via chemical or physical means and this ultimately influence cell behaviour and tissue bio-integration. Whilst surface modification techniques such as UV/ozone and oxygen plasma activation are rapid and easy to use, they suffer from hydrophobic recovery. Current approaches overcome hydrophobic recovery through combining different modification techniques, in most cases involving a pre-treatment stage followed by a coating step. Natural polymers are an excellent choice for coating silicone surfaces as they mimic the natural ECM but suffer from lack of tensile strength. Synthetic polymers display great mechanical properties but suffer from lack of functional groups needed for interactions with cells and tissue. As shown in this review, recent work show that a combination of both natural and synthetic polymers may be the best way to overcome various challenges.

The work presented here demonstrates that following the insertion of a silicone implant into the breast cavity, the breast tissue undergoes repair. Soon after implant insertion the host tissue responds by the formation of a provisional matrix, initiation of acute inflammation, and finally fibrous capsule formation. Body fluids such as blood and wound fluid would soak the silicone implant first. Proteins within these fluids adsorb onto the implant surface. Typical proteins include vitronectin, collagens, albumin, and fibrinogen. The layer of proteins adsorbed onto the implant surface and the conformation of these proteins are influenced by implant surface properties such as surface topography and the hydrophilicity or hydrophobic nature of the implant surface. In addition, the adhesion of cells to the implant occurs via integrins interactions with the layer of proteins adsorbed on the implant.

Theories suggest that conditions such as ALCL may be caused by bacterial infection. Functionalisation of silicone implant surfaces via the use of UV/ozone and UV irradiation treatments adds reactive species on the surface of the implants rendering it unhabitable to bacteria. Coating the surfaces of silicone implants with various ECM proteins to modulate FBR and prevent pathological conditions such as ALCL is being investigated by various laboratories worldwide. In addition, the coating of silicone implant surfaces with nanoparticles is being investigated. New technologies including 3D printing can be harnessed to produce silicone surfaces with the right topography to modulate FBR and capsule formation after insertion of the implant.

## Conclusions

Due to the inertness and other physical properties of silicone material, silicone implants may not integrate into the human body as well as other materials. To attach to the silicone implant, cells synthesize and release several biomolecules which adsorb onto the silicone surface, allowing cells to eventually bind to the implant. These adsorbed molecules thus influence gene expression, adhesion, proliferation, and spread of cells on the silicone surface. Several properties of the surface such as its roughness, wettability, and stiffness influence cellular adhesion and proliferation. Unmodified silicone material is highly hydrophobic and demonstrate poor cell adhesion and tissue biointegration. In turn, the amount and the conformation of the biomolecules adsorbed on the silicone implant surface can play a key role in determining the strength of cellular attachment to the silicone implant. The use of bio-mimetic engineered surfaces in addition to the presence of key biomolecules can help prevent complications such as capsular contracture and anaplastic large cell lymphoma formation. Future studies are needed to further study the influence of bio-inspired silicone implant surface modifications and their fate *in vivo*.

## Data Availability

Data sharing is not applicable to this article as no datasets were generated or analysed during the current study.

## Abbreviations

3D: Three-dimensional
AA: Alginic acid
ALCL: Anaplastic large cell lymphoma
αMβ2: Integrin alpha M beta-2
αVβ1: Integrin alpha V beta-1
CCL2: Chemokine C–C motif ligand 2
CD: Cluster of differentiation
CMC: Carboxymethyl cellulose
CMD: Carboxymethyl -1,3-dextran
CXCL10: Chemokine C-X-C motif chemokine ligand 10
DAMPs: Damage-associated molecular patterns
E: Elastic modulus
ECM: Extracellular matrix
FBR: Foreign body response
FBGCS: Foreign body giant cells
FDA: Food and Drug Administration
HA: Hyaluronic acid
IgG: Immunoglobulin G
IL: Interleukin
ISAPS: International Society of Aesthetic Plastic Surgery
ISO: International Organization for Standardization
MCP-1: Monocyte chemoattractant protein-1
MIP-1a: Macrophage inflammatory proteins 1a
MKN: Nε-myristoyl-lysine methyl ester
MMP: Matrix metallopeptidase
NET: Neutrophil extracellular traps
OC: Open cavities
OEG: Oligo (ethylene glycol)
OEGMA: Oligo (ethylene glycol) methyl ether methacrylate
PAAm: Poly(acrylamide)
PC: Phosphorylcholine
pCBMA: Poly carboxybetaine methacrylate
PDGF: Platelet-derived growth factor
PDMS: Polydimethylsiloxane
PEG: Poly (ethylene glycol)
PEG-SBSi: Polyethylene glycol sulfobetaine silane
PEG-silane: Methoxy(polyethyleneoxy) propyltrimethoxysilane
PF4: Platelets secrete platelet factor 4
PGA: Poly-glycolic acid
PHEMA: Poly (hydroxyethyl methacrylate)
PHSRN: Proline-histidine-serine-arginine-asparagine
PLA: Poly-lactic acid: PLGA: poly-lactic-co-glycolic acid
PLL: Poly-l-lysine
PMAA: Poly (methacrylic acid)
PMNs: Polymorphonuclear leukocytes
PMPC: Poly (2-methacryloyloxyethyl phosphorylcholine)
polyNaSS: Poly (Sodium Styrene Sulfonate)
PV: Peak and valley
PVA: Polyvinyl alcohol
PVP: Polyvinylpyrrolidone
qPCR: Quantitative polymerase chain reaction
Ra: Arithmetic mean height
RAFT: Reversible addition-fragmentation chain transfer
RGD: Arginine–glycine–asparagine
SBSi: Sulfobetaine silane
Sku: Kurtosis value
SOC: Semi-opened cavities
Ssk: Surface skewness
TGF-β: Transforming growth factor beta
TIMP4: Metalloproteinase inhibitor 4
TNF-α: Tumour necrosis factor α
TNSFS11: Tumour necrosis factor ligand superfamily member 11
WHO: World Health Organization
