# Correction: Advances in surface modifications of the silicone breast implant and impact on its biocompatibility and biointegration

**DOI:** 10.1186/s40824-023-00437-z

**Published:** 2023-10-02

**Authors:** Fatemeh Tavakoli Foroushani, Kevin Dzobo, Nonhlanhla P Khumalo, Vanessa Zamora Mora, Roberto de Mezerville, Ardeshir Bayat

**Affiliations:** 1 Wound and Keloid Scarring Research Unit, Hair and Skin Research Laboratory, Division of Dermatology, Department of Medicine, The South African Medical Research Council, University of Cape Town, Cape Town, South Africa; 2 Establishment Labs Holdings, Roberto de Mezerville Establishment Labs Holdings, Alajuela, Costa Rica

**Biomaterials Research (2022) 26:80**

10.1186/s40824-022-00314-1

Vanessa Zamora Mora and Roberto de Mezerville are salaried employees of the Establishment Labs company. Ardeshir Bayat is a co-inventor on a number of patents that were assigned to the Establishment Labs company and he is also a financially compensated member of the scientific advisory board of the same company.

